# The new frontier in assisted reproduction: Consumer Desire vs. Regulatory and Ethical Precaution in AI-assisted Polygenic Embryo Screening

**DOI:** 10.1038/s44319-025-00668-2

**Published:** 2025-12-10

**Authors:** Aviad Raz, Aurélie Halsband, Robert Langner, Shiri Shkedi-Rafid

**Affiliations:** 1 Dept. of Sociology and Anthropology, Ben-Gurion University of the Negev, Beer-Sheva, Israel; 2 German Reference Centre for Ethics in the Life Sciences (DRZE), University of Bonn, Bonn, Germany; 3 Institute of Systems Neuroscience, Medical Faculty and University Hospital, Heinrich Heine University Düsseldorf, Düsseldorf, Germany; 4 Institute of Neuroscience and Medicine (INM-7: Brain and Behaviour), Research Centre Jülich, Jülich, Germany; 5 Department of Genetics, Hadassah Medical Center, Faculty of Medicine, Hebrew University of Jerusalem, Jerusalem, Israel

**Keywords:** Development, Economics, Law & Politics, Genetics, Gene Therapy & Genetic Disease

## Abstract

AI-assisted polygenic embryo screening is a new technology to predict embryonic implantation, disease risk and non-clinical traits for IVF embryos. To highlight its social and ethical implications, we locate it within the national landscapes of Germany’s legal caution, Israel’s techno-enthusiasm and US market liberalism.

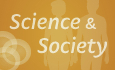

In 2023, the US company Orchid Health announced artificial intelligence (AI)-assisted polygenic embryo screening (PES) as a major innovation in preimplantation genetic testing (PGT) for polygenic disorders. Since the 1990s, users of in vitro fertilization (IVF) have been able to genetically test embryos for single, mainly severe, highly penetrant diseases such as Tay-Sachs, Duchenne Muscular Dystrophy, or Cystic Fibrosis. Technologies that improved the success rate of IVF were thus combined with opportunities for genetic testing, often referred to as *reprogenetic technologies*.

The new AI-based technology enables the screening of embryos for additional complex conditions and traits, the development of which is influenced by many genetic variants, beyond the scope of current testing methods. It builds on polygenic risk scoring (PRS), a probabilistic method that aggregates small genetic effects across the genome to estimate the risk of complex pathologies such as diabetes, cancer, or cardiovascular diseases. The utility and implications of PRS, however, remain highly contested (Raz and Minari, 2023). While AI-assisted PES has been celebrated as a new frontier of reproductive choice in the US media, critics have warned of its uncertain validity, potential for misuse, and the slippery slope of genetic selection. These tensions highlight a growing gap between consumer desire and ethically informed precautions in reproductive innovation.

The new AI-based technology enables the screening of embryos for additional, complex conditions and traits […] beyond the scope of current testing methods.

## AI-driven improvements

AI-assisted PES could enhance embryo selection for IVF in three main ways. First, it allows the evaluation of the embryo’s likelihood of being euploid—possessing the correct number of chromosomes—thereby reducing the risks for relatively common conditions such as Down syndrome. This option also enables grading the embryos’ potential for developing into a fetus after implantation in the womb. AI algorithms already outperform human embryologists in selecting viable embryos for implantation (Salih et al, 2023). Such advances suggest that AI-assisted PES could improve IVF success rates and reduce costs by enabling more efficient embryo selection. Second, it can estimate polygenic risks for late-onset medical conditions such as cardiovascular disease, diabetes, or cancer (Haining et al, 2025). Third, and most controversially, it can be extended to the prediction of non-medical traits such as height or intelligence (Davies et al, 2018).

However, such non-medical trait predictions are probabilistic and inherently uncertain, particularly because polygenic scoring captures only genetic contributions while neglecting environmental and epigenetic factors. Yet, recent AI-based approaches promise to improve predictive accuracy by extending the feature space and integrating large-scale genomic, clinical, and environmental factors (Khanna et al, 2023; Hosseini et al, 2025).

Beyond issues of performance accuracy and predictive value, AI-assisted PES raises profound ethical and social questions. It magnifies long-standing concerns about eugenics and the commodification of reproduction. Similar to earlier debates on preimplantation testing, the new technology challenges existing moral and regulatory frameworks by promising greater control over reproduction while deepening inequalities in access and amplifying societal pressures to conform to genetic ideals. The potential benefits—greater success, accuracy, efficiency, and personalization—must therefore be weighed against risks of discrimination, inequity, and loss of reproductive autonomy.

Similar to earlier debates on preimplantation testing, the new technology challenges existing moral and regulatory frameworks…

## National contexts: Germany, Israel, and the USA

A comparative analysis is essential for understanding the contemporary social and regulatory landscapes of AI-assisted PES. Germany and Israel, both with socialized healthcare systems, offer contrasting models of how reproductive technologies are regulated and culturally perceived. The USA, by contrast, represents a privatized, market-driven system where IVF operates largely outside national regulation. These cases together illuminate how cultural values, historical legacies, market forces, and healthcare shape the governance and acceptance of new reproductive technologies.

## Germany: restrictive regulation and the lessons of history

In Germany, the regulation of IVF and related technologies are shaped by the legacy of Nazi eugenics and legal frameworks that strongly emphasize the protection of the embryo and human dignity. Germany’s legal framework is unique, as fetal anomaly itself is not a sufficient legal ground to terminate a pregnancy. Equally, the regulation of prenatal screening programs puts a strong emphasis on embryo protection. This contrasts with many other countries where the severity of fetal conditions is a central criterion for the regulation of reproductive treatments. Overall, the German Embryo Protection Act (Embryonenschutzgesetz) regulates reproductive medicine strictly: PGT is only permitted for severe hereditary diseases and requires prior ethics approval. PES is currently prohibited.

**…** the German Embryo Protection Act (Embryonenschutzgesetz) regulates reproductive medicine strictly: PGT is only permitted for severe hereditary diseases and requires prior ethics approval.

Public opinion, however, is more nuanced. Surveys show that while most Germans support PGT for preventing serious diseases, they strongly oppose its use for enhancement or trait selection (Meyer et al, 2023). Another study of public attitudes toward PGT in Germany (Arzheimer, 2020) showed how they diverge from the restrictive legislation, with widespread secular support for allowing PGT not just for severe genetic diseases—as is permitted under special conditions by the Präimplantationsdiagnostikgesetz from 2011—but also for any genetic disease. It is indeed intriguing that the German legal acknowledgement of severity as a criterion for selective prevention takes place in the extra-corporeal context of PGT—extra-corporality being defined in relation to the woman’s body—but not during pregnancy.

The German media, however, still often frame PES through cautionary narratives referring to “designer babies” and slippery slopes toward genetic enhancement. Media reports like MDR Wissen’s *Babies on demand: Embryo screening for smarter children?* point to fears of parental pressure and threats to the self-determination of future children. Academic and professional discourses largely mirror these reservations: the German Ethics Council and the European Society of Human Genetics have called PES “unproven” and “ethically problematic,” emphasizing the need for substantial scientific validation before clinical use (Forzano et al, 2022). Despite growing calls from German medical associations to modernize reproductive laws, the prevailing ethical stance remains one of precaution, grounded in Germany’s historical sensitivity to reproductive selection and its moral emphasis on protecting human life at the earliest stage.

## Israel: innovation, technology acceptance, and consumer orientation

Israel presents a strikingly different case. Regulation of PGT in Germany and Israel arguably relates to two different paradigms of responsibility: toward the protection of embryos in Germany and toward procreative autonomy in Israel (Hashiloni-Dolev and Shkedi, 2007). This difference also reflects a gradualist approach to the moral status of the (pre)embryo at the beginning of life, which, in Israel, is not perceived as an all-or-nothing construction (ESHRE, 2024).

With one of the world’s highest per capita rates of IVF and widespread acceptance of reproductive genetic testing, Israel’s cultural and medical environment is uniquely receptive to innovations in the fields of fertility and genetics. While no explicit legal framework currently governs PES, preimplantation testing for diseases is funded, under specific guidelines, by the state, including for creating “donor siblings,” that is, human leukocyte antigen matching of an existing child who suffers from a life-threatening, monogenic disease that requires bone-marrow transplantation for treatment. Generally, Israel is a country where new reprogenetic technologies tend to be rapidly integrated into practice. The Israeli media have depicted PES as an inevitable next step in the high-tech IVF journey, with headlines such as *Designer babies? Hi-tech preimplantation genetic testing may soon come to Israel*. Israeli researchers, such as Lencz et al, 2021, have emphasized both the potential and limits of PES, showing that its utility depends on embryo numbers and polygenic score strength. The Israeli context demonstrates how a pro-natalist culture, state-supported IVF and a techno-optimistic ethos together can foster rapid adoption of emerging reproductive technologies.

The Israeli context demonstrates how a pro-natalist culture, state-supported IVF and a techno-optimistic ethos together can foster rapid adoption of emerging reproductive technologies.

## The USA: privatization, market logic, and limited oversight

The USA represents a distinct, although instructive case. IVF and related services operate almost entirely in the private sector, with limited insurance coverage and minimal federal regulation. Clinics are governed primarily by professional guidelines rather than statutory oversight, leading to wide variation in practices. Many IVF screening programs are owned and offered by profit-oriented private companies, making them very aggressive in marketing with no real regulation of ads or marketing claims. Moreover, US liability laws may fuel both supply and demand as doctors fear being sued for failure to offer the best available screening. This market-driven environment fosters rapid technological adoption but also commercial competition that can outpace ethical review. The launch of AI-assisted PES by Orchid Health exemplifies this dynamic: it is marketed as empowering consumer choice, yet lacking robust validation or regulatory control. Surveys indicate strong public approval though: around 72% of respondents support PES in principle, and 82% would consider it if already undergoing IVF. However, support for PES to screen for non-medical traits is much lower at 30% (Furrer et al, 2024).

When IVF is largely treated as a private business rather than a public-health service, ethical debates are often reframed in terms of consumer choice, focusing on personal freedom, innovation, and market differentiation rather than collective responsibility or distributive justice. Consequently, the US system may be too different on various dimensions for direct comparisons with Israel or Germany where healthcare systems are socialized, and regulation is centralized. Nonetheless, the US model highlights the challenges of unregulated innovation, where market demand may override precautionary ethics, potentially globalizing commercial norms that challenge more restrictive systems elsewhere (Barlevy et al., 2024).

**…** the US model highlights the challenges of unregulated innovation, where market demand may override precautionary ethics…

## From prevention to enhancement

The promised advantages of AI-assisted PES include increased accuracy in risk prediction, improved embryo selection, potentially lower costs due to automation and efficiency gains, and overall higher success rates in reproductive treatments (Khanna et al, 2023; Salih et al, 2023). AI-based prediction models using a combination of medical images and clinical information have a median accuracy of 81.5% in predicting IVF pregnancy, significantly higher than the 51% median accuracy of clinical embryologists (Salih et al 2023). If these efficiencies translate into higher pregnancy rates, the technology could reduce the financial and emotional burdens for patients. Furthermore, AI’s capacity to integrate genomic and phenotypic data could enhance personalized reproductive medicine, tailoring embryo selection to optimize health outcomes. Such potential benefits appeal strongly to both clinicians and patients/clients seeking better results from financially and emotionally costly IVF cycles.

Beyond predictive screening of embryos, AI-optimized PRS models, compared to non-AI models, were found to enhance predictive accuracy in cardiology for adult patients by improving feature selection, handling high-dimensional data, and integrating diverse variables, enabling more personalized prevention strategies (Hosseini et al, 2025). However, PRS calculated from genetic association studies cannot fully account for the influence of environmental factors in late-onset conditions when most data available for embryos is genetic. In that sense, the promise of AI-driven integration of clinical, environmental, and genetic data for better predictions of PRS has little evidence base in the case of embryos.

We argue that while AI-assisted PES does not essentially change the ethical debate, the introduction of AI in reproductive medicine in general may well push the regulatory boundaries in a manner that requires additional ethical discussion. For example, if the option of AI-assisted IVF enables grading the IVF embryos’ potential for better implantation and development, should we still prohibit it under embryo protection considerations? This is not just a specific issue of AI-assisted PRS integration into embryo screening, but also a more general example of AI’s integration into reproductive medicine.

Soon, AI-assisted embryo scoring for improving IVF success will probably be offered together with AI-assisted PES. We need empirical work modeling how much benefit PES might deliver. However, we also need reflective ethical work to understand how such new technologies push the current evaluative boundaries (Halsband, 2025), as well as empirical work to learn how such pushes would be perceived and evaluated by the people.

The advances of PES are counterbalanced by serious ethical, social, and regulatory challenges. First, the probabilistic nature of PES means that even “high-risk” or “low-risk” embryos are subject to uncertainty; false expectations could lead to psychological distress or misplaced trust in genomic data (Haining et al, 2025). Second, widespread adoption could generate new forms of social pressure: if PES becomes normalized, parents might feel obliged to use it to secure the “best” genetic start for their children. This could undermine reproductive autonomy and reinforce narrow definitions of normality and success. Third, AI systems are prone to algorithmic bias, particularly if trained on non-representative—for instance, insufficiently diverse—genomic datasets, potentially exacerbating health disparities and excluding minority populations (Raz and Minari, 2023). Fourth, issues of equal access loom large: in privatized systems such as the USA, such technologies may be available only to affluent patients, while public systems have to face questions of prioritization and resource allocation. Finally, PES revives long-standing fears of eugenics and genetic enhancement, albeit now reframed in the language of consumer choice rather than ideology-driven state coercion.

Ethically, the debate over PES continues and magnifies earlier controversies over IVF, PGT, and prenatal testing. Each successive innovation has expanded the scope of choice while shifting the moral boundaries of what counts as ethically acceptable reproduction technologies. PES, with its promise of AI-enhanced precision and predictive reach, pushes these boundaries further by linking genomic data to probabilistic futures and by blurring the line between disease prevention and selection for enhancement. Where earlier reproductive technologies were justified by the desire to avoid severe disorders, PES risks transforming reproduction into optimization. Thus, the introduction of AI into embryo selection does not fundamentally change the ethical terrain—it merely intensifies it. It calls for renewed reflection on autonomy, equity, and the moral meaning of genetic selection in a world increasingly shaped by algorithmic reasoning.

**…** the introduction of AI into embryo selection does not fundamentally change the ethical terrain—it merely intensifies it.

## Conclusions

AI-assisted PES stands at the intersection of technological optimism and ethically rooted caution. It offers the promise of more accurate, efficient, and individualized reproductive choices but simultaneously revives concerns about social inequality, genetic normalization, and the commodification of life. This intersection becomes even more complicated as implantation prediction, disease-risk prediction, and evidently prediction of nonclinical traits, such as intelligence or hair color, all carry different ethical concerns depending on their risks and benefits. For example, the risks in grading to identify “less potent” IVF embryos may be seen as ethically less controversial than using PES for trait selection, which raises the specter of eugenic selection/enhancement and genetic discrimination. As such, inter-connected discussions on ethics and regulation of AI-assisted PES need to consider how this evolving technology is used, what reasons it is used for, who gets to use it, and who pays for it.

Our summary of the contrasting national landscapes of the USA, Israel, and Germany illustrates the cross-national variety of policies, with no current regulatory model being able to resolve all the tensions: Germany’s legal caution, Israel’s techno-enthusiasm, and the United States’ market liberalism each reflect broader cultural and institutional values. Cross-national comparison also underscores that public attitudes do not always align with regulatory traditions: Permissive views in Israel coexist with a current lack of medical-legal guidelines (which might change soon), while German (secular) publics arguably often express greater acceptance of embryo testing than is currently permitted by law. As AI continues to reshape reproductive medicine, ethical frameworks may have to evolve beyond national borders, integrating empirical data with normative reflection. Ultimately, the debate over AI-assisted PES is not only about what we can predict or select but about what kind of future we wish to design.

Ultimately, the debate over AI-assisted PES is not only about what we can predict or select but about what kind of future we wish to design.

## Supplementary information

Peer Review File
