# Supplementary material for: The new frontier in assisted reproduction: Consumer Desire vs. Regulatory and Ethical Precaution in AI-assisted Polygenic Embryo Screening
Source: EMBO Rep. 2025 Dec 10;27(2):265–8. doi: 10.1038/s44319-025-00668-2 (PMC12852876; doi:10.1038/s44319-025-00668-2)
Supplement: Supplementary file 1 — Peer Review File [file 44319_2025_668_MOESM1_ESM.pdf]

EMBO reports

The new frontier in assisted reproduction. Consumer Desire vs. Regulatory and Ethical Precaution in AI-assisted Polygenic Embryo Screening

Aviad Raz, Aurélie Halsband, Robert Langner, and Shiri Shkedi-Rafid

Corresponding author(s): Aviad Raz (aviadraz@bgu.ac.il)

Review Timeline:

Submission Date:	10th Nov 25
Accepted:	26th Nov 25

Editor: Holger Breithaupt

Transaction Report: This article was reviewed with board member advice.
